# Surface Energy of Au Nanoparticles Depending on Their Size and Shape

**DOI:** 10.3390/nano10030484

**Published:** 2020-03-08

**Authors:** David Holec, Phillip Dumitraschkewitz, Dieter Vollath, Franz Dieter Fischer

**Affiliations:** 1 Department of Materials Science, Montanuniversität Leoben, Franz Josef Straße 18, A-8700 Leoben, Austria; 2 Chair of Nonferrous Metallurgy, Department of Metallurgy, Montanuniversität Leoben, Franz-Josef-Straße 18, A-8700 Leoben, Austria; phillip.dumitraschkewitz@unileoben.ac.at; 3 NanoConsulting, Primelweg 3, D-76297 Stutensee, Germany; dieter.vollath@nanoconsulting.de; 4 Institute of Mechanics, Montanuniversität Leoben, Franz Josef Straße 18, A-8700 Leoben, Austria; mechanik@unileoben.ac.at

**Keywords:** surface energy, nanoparticles, gold, ab initio, molecular mechanics

## Abstract

Motivated by often contradictory literature reports on the dependence of the surface energy of gold nanoparticles on the variety of its size and shape, we performed an atomistic study combining molecular mechanics and ab initio calculations. We show that, in the case of Au nanocubes, their surface energy converges to the value for (001) facets of bulk crystals. A fast convergence to a single valued surface energy is predicted also for nanospheres. However, the value of the surface energy is larger in this case than that of any low-index surface facet of bulk Au crystal. This fact can be explained by the complex structure of the surface with an extensive number of broken bonds due to edge and corner atoms. A similar trend was obtained also for the case of cuboctahedrons. Since the exact surface area of the nanoparticles is an ill-defined quantity, we have introduced the surface-induced excess energy and discuss this quantity as a function of (i) number of atoms forming the nano-object or (ii) characteristic size of the nano-object. In case (i), a universal power-law behaviour was obtained independent of the nanoparticle shape. Importantly, we show that the size-dependence of the surface energy is hugely reduced, if the surface area correction is considered due to its expansion by the electronic cloud, a phenomenon specifically important for small nanoparticles.

## 1. Introduction

Surface energy is an important thermodynamic quantity. Particularly in cases where the volume-to-surface ratio becomes small, as is the case of nanoparticles, its relevance must not be underestimated [1,2].

There has been a vivid discussion concerning the qualitative trend of the surface energy as a function of the nanoparticle size. On the one hand, in many cases one finds reports on decreasing surface energy with decreasing particle size, e.g., in a study by Vollath and Fischer [3] or earlier studies [4,5]. This trend has been conventionally explained with an increasing tendency to form a liquid-like structure at the surface of the particles [6]. On the other hand, there exists a number of primarily theoretical papers finding a significant increase of the surface energy with decreasing particle size, see, e.g., Refs. [7,8,9]. Furthermore, there are also some heavily disputed experimental results indicating an increasing surface stress (and hence, due to a conventional assumption, also surface energy) with decreasing particle size [10,11]. Nanda et al. [11] pointed out that the difference between various reported trends stems from the nanoparticle nature. The surface energy is expected to increase for free nanoparticles with decreasing particle size, while the opposite trend is obtained for nanoparticles embedded in a matrix.

Wei and Chen [12] pointed out that, from the theoretical point of view, the trend could be qualitatively altered by changing the definition of a nanoparticle surface area. Unlike the energy change related to forming the free surface of a nanoparticle, the area is not well defined. Consequently, small changes of the radius/size yield large changes of the surface area, especially for nanometre-sized particles [12]. The rather geometrical argumentation of Ref. [12] was later linked to a physical quantity, a spatial expansion of the electronic cloud [13]. Using a refined, physically-based surface for small nanoparticles consequently leads to a weak-to-no size dependence of surface energy [14,15]. The latter reference also provided a thermodynamical-based model with predictive capabilities, hence seemingly resolving the enigma regarding the size dependence of the surface energy.

Nanoparticles, and particularly gold nanoparticles, nonetheless present a rich area of application as well as curiosity-driven research. Their applications span from biomimetic materials, over printed electronics to electrochemical biosensors [16,17]. Quite counterintuitively, the most preferable structure of a 55 Au atoms cluster was shown to be an amorphous structure even at 0K [18], being a consequence of the small nanoparticle size. This prediction, however, was experimentally corroborated [18]. Ali et al. [9] predicted a rapid increase of the surface energy upon the nanoparticle melting. In agreement with earlier work of Shim et al. [19], they also predicted the decrease of melting temperature with decreasing nanoparticle size. Spontaneous segregation to some facets has been reported for Au-Ni nanoparticles, leading to an overall isotropic elastic response [20]. Another interesting effect is the shape variety of nanoparticles, accessible via solution synthesis modifying the surface energy in its very essence [17,21].

In the present study we, therefore, employ atomistic simulations to study the impact of nanoparticle shape on the resulting surface energy estimation. We focus on shapes ranging from rather artificial but geometrically simple nanocubes, over cuboctahedrons (special members of the truncated octahedrons, which have been reported as equilibrium shapes of Au nanoparticle), to nanospheres. In the final section we discuss how is the shape and size dependence of the surface-induced excess energy (i.e., the total nanoparticle surface energy) are related to number of broken bonds due to the creation of the free surface.

## 2. Methodology

Molecular mechanics (MM) simulations were performed using the LAMMPS package [22] together with an interatomic potential describing the gold interatomic interaction within the embedded atom method (EAM) as parametrised by Grochola et al. [23]. The individual idealised nanoparticles with well-defined shapes were cut out from bulk fcc structure with lattice constants of 4.0694Å. This was obtained from fitting calculated total energies corresponding to different bulk volumes with Birch-Murnaghan equation of state [24], and agrees well with the values 4.0701Å obtained by Grochola et al. [23]. All models were structurally relaxed using conjugate-gradient energy minimisation scheme at 0K with force-stopping convergence criterion set to $10^{-12}\;\mathrm{eV}/Å$.

Additionally, a few ab initio runs were performed to benchmark our MM calculations. We used Vienna Ab initio Simulation Package (VASP) [25,26] implementation of Density Functional Theory (DFT) [27,28]. Two common approximations of the electronic exchange and correlation effects were considered: local density approximation (LDA) [28] and the Perdew–Wang parametrisation of the generalised gradient approximation (GGA) [29]. The contribution of ions and core electrons were described by projector augmented wave (PAW) pseudopotentials [30]. The plane wave cut-off energy was set to 400eV, and the reciprocal space sampling was equivalent to 10×10×10 *k*-mesh for the fcc-conventional cell. In directions, where periodicity should be avoided (e.g., the direction of the slab, all 3 directions in the case of nanoparticles), only a single *k*-point was used. In other directions, the number of *k*-points was scaled so that the *k*-point spacing in the reciprocal space was kept constant, i.e., ≈π/(10·4.069) Å${}^{-1}=0.077$ Å${}^{-1}$, where 4.069 Å is the lattice parameter of fcc-Au. Due to the employed periodic boundary conditions, we used a simulation box ≈20Å larger than the actual (unrelaxed) nanoparticle to avoid any undesired interactions through the vacuum separating neighbouring nanoparticles. Similarly, ≈15Å vacuum in the direction perpendicular to a free surface was used to separate slabs for calculating the surface energies of bulk Au. The electron charge was considered converged when the total energy of two subsequent self-consistency cycles differed by less than $10^{-4}\;\mathrm{eV}$, whereas structural optimisations were stopped when the total energy of two subsequent configurations differed by less than $10^{-3}\;\mathrm{eV}$. These criteria provide a total energy accuracy in the order of 1meV/at. or better.

Finally, the qhull program [31] was used to calculate an area of a convex hull of ionic positions for each nanoparticle, to be used as an estimate of the surface area.

## 3. Results

### 3.1. Low-Index Facets of Bulk Au

The results presented in this chapter serve the subsequent discussion of the MM results, and their accuracy with respect to first principles calculations. Surface energy, γ, of a surface facet (hkl) can be calculated as

(1) $$\gamma =\frac{1}{2A}\left(E_{\mathrm{slab}}-NE_{\mathrm{bulk}}\right)\;,$$

where $E_{\mathrm{slab}}$ is energy of a slab composed of *N* layers. $E_{\mathrm{bulk}}$ is the energy of the bulk material per one layer of cross-section *A*. The factor 2 results from the fact that the slab has two surfaces. A layer is understood as a surface primitive cell, i.e., when the desired facet (hkl) is perpendicular to one of the lattice vectors (for a detailed description of the surface primitive cells, see e.g., Ref. [32]). Due to the interaction of the two free surfaces, either through the vacuum (i.e., not well separated slabs in the case of periodic boundary conditions) or the bulk of the slab (i.e., too thin slab), the value γ has to be converged with respect to both of these. In the case of MM simulations, only the latter convergence needs to be tested if the simulation is run in a box without periodic boundary conditions in the direction perpendicular to the free surface.

Test calculations revealed that vacuum of 10Å is sufficient to get surface energy results converged to well below $1\;\mathrm{meV}/{Å}^{2}$. Similarly, a slab thickness of about 40Å is needed in order to avoid interactions of the free surfaces through the gold layer. The obtained values from the DFT benchmarks and MD simulations are summarised in Table 1. The here obtained DFT values are comparable with data from the literature. They exhibit the same ordering ($\gamma_{\left(1\;1\;0\right)}>\gamma_{\left(1\;0\;0\right)}>\gamma_{\left(1\;1\;1\right)}$) as reported earlier [33]. In a simplified picture, the surface energy expresses energy penalty related to the areal density of broken bonds [32,34]. This is $8/a_{0}^{2}$ for the (100) surface, $7.07/a_{0}^{2}$ for (110), and $4.33/a_{0}^{2}$ for the (111) surface ($a_{0}$ being the fcc lattice constant). The density of broken bonds is similar for the (100) and (110) surfaces, while it is significantly lower for the (111) orientated facet, hence providing a qualitative explanation for the surface energy ordering.

The DFT and MM values exhibit an almost constant difference between the corresponding surface energies. Moreover, the MM values are very close to the DFT-LDA results. This is a somewhat surprising result since the EAM potential has been fitted to the DFT-GGA data using the same parametrisation by Perdew and Wang [29] as used here. We speculate that this is caused by fixing 4.07Å as the lattice constant during the EAM potential fitting [23], as our LDA and GGA calculations yielded 4.061 and 4.176Å, respectively. Nevertheless, since LDA and GGA are known to overestimate and underestimate, respectively, binding [38], and since the MM values are in between the two DFT-based estimations, we conclude that the interatomic potential used here is suitable for studying trends in surface energies. Moreover, the resulting values are expected to be very close to DFT-LDA calculations.

### 3.2. Impact of Shape and Size on the Nanoparticles Surface Energy

The surface energy of a gold nanoparticle consisting of *N* atoms is defined as an excess energy with respect to the energy of *N* atoms of bulk fcc gold, normalised to the nanoparticle surface area, *A*:

(2) $$\gamma =\frac{E_{\mathrm{nanoparticle}}-NE_{\mathrm{bulk}}}{A}.$$

In the above, $E_{\mathrm{nanoparticle}}$ is the total energy of the nanoparticle, while $E_{\mathrm{bulk}}$ is energy per atom of bulk fcc Au. Unlike the total energies, the surface area *A* is not a well defined quantity. In the following sections, an area of a convex hull of the relaxed ionic positions is consistently used as an estimate for *A*.

#### 3.2.1. Nanocubes

**Figure 1:**
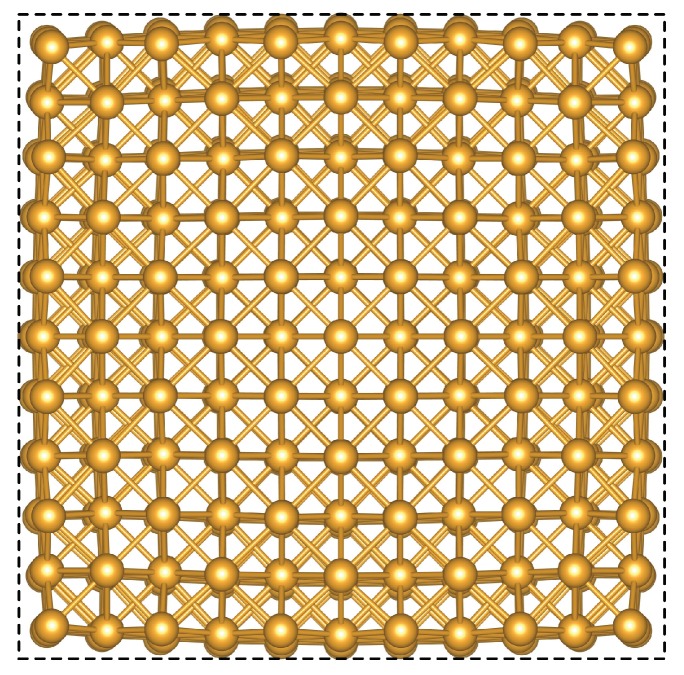
Relaxed structure of a nanocube with side a=2.035nm (666 atoms). The dashed line is a guide for the eye showing an ideal square shape.

In order to calculate the total energy of {100}-faceted nanocubes, structural models with a side length up to 20nm were fully structurally relaxed. As a consequence of the surface tension, the apexes “popped in” as is apparent from the snapshot of relaxed atomic positions shown in Figure 1.

**Figure 2:**
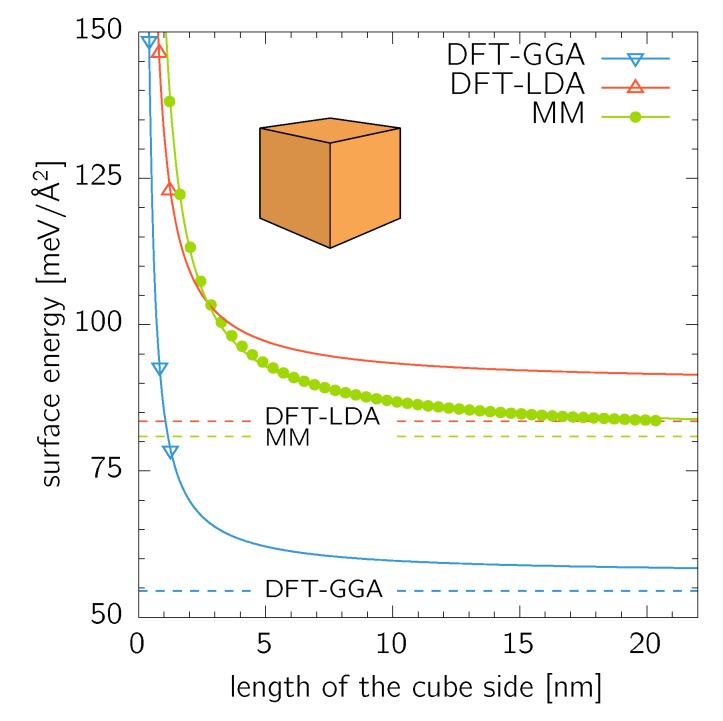
Surface energy of nanocubes calculated by DFT and MM. The calculated datapoints were fitted with Equation (3). The dashed lines are (100) surface energies as listed in Table 1.

Supercells up to 3×3×3 conventional fcc cell (172 atoms) were treated using the DFT, while nanocubes up to 50×50×50 (515 151 atoms) were calculated using MM. A nanocube formed from n×n×n conventional cubic fcc cells (4 atoms per cell) contains $N=4n^{3}+6n^{2}+3n+1$ of atoms. The calculated surface energy values shown in Figure 2 were fitted with an exponential relationship

(3) $$\gamma =\gamma_{0}\exp\left(\frac{L}{a}\right),$$

where $a=n\cdot a_{0}$ is the side length of a cube formed by n×n×n conventional fcc cells with the lattice parameter $a_{0}$. The quantities $\gamma_{0}$ and L are used as two fitting parameters. The thus obtained values of the pre-exponential parameter, $\gamma_{0}^{\mathrm{GGA}}=57.4\;\mathrm{meV}/{Å}^{2}$, $\gamma_{0}^{\mathrm{LDA}}=89.8\;\mathrm{meV}/{Å}^{2}$, and $\gamma_{0}^{\mathrm{MM}}=81.4\;\mathrm{meV}/{Å}^{2}$ agree well with the bulk surface energies for the (100) facets ($\gamma_{\left(1\;0\;0\right)}^{\mathrm{GGA}}=54.5\;\mathrm{meV}/{Å}^{2}$, $\gamma_{\left(1\;0\;0\right)}^{\mathrm{LDA}}=83.5\;\mathrm{meV}/{Å}^{2}$, and $\gamma_{\left(1\;0\;0\right)}^{\mathrm{MM}}=80.9\;\mathrm{meV}/{Å}^{2}$). This is an expected result as the bulk values are limits for infinitely large cubes. It is, however, surprising, that such a good agreement is obtained for the DFT data where only three data points are available for the fitting procedure. The same fitting procedure yielded for the parameter L (Equation (3)) values of 0.397nm, 0.392nm, and 0.061nm for DFT-GGA, DFT-LDA, and MM data sets, respectively.

#### 3.2.2. Nanospheres

**Figure 3:**
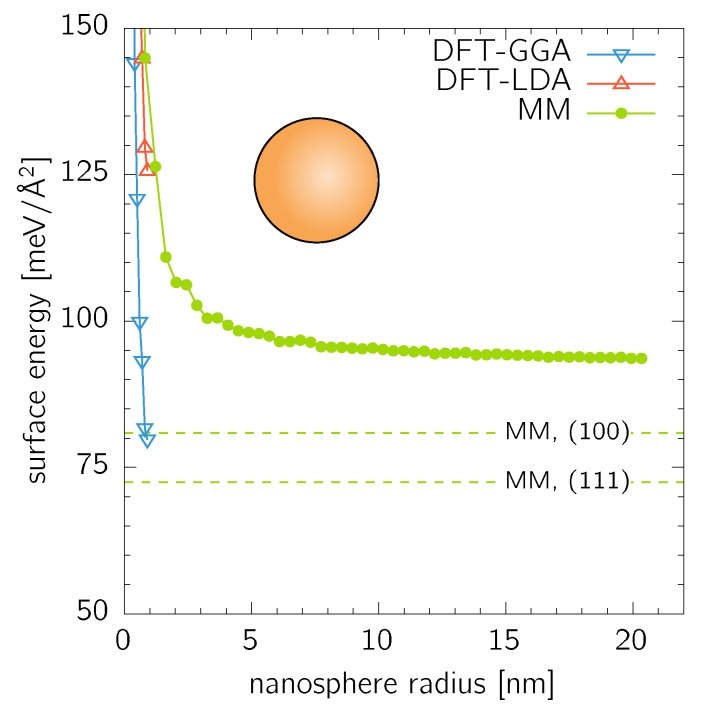
Surface energy of nanospheres calculated by DFT and MM. The dashed lines are the MM values for single-orientated (100) and (111) surfaces as listed in Table 1.

Nanospheres with all possible facet orientations were considered as an opposite extreme to the nanocubes with only a single orientation of their facets. They were constructed by cutting material contained in an ideal sphere of a given radius out of an infinitely large fcc Au crystal. The DFT calculations were performed up to r=0.9nm (152 atoms), while the MM calculations allowed easily for spheres up to r=20.3nm (2 094 177 atoms) (Figure 3). In comparison to the case of nanocubes, the surface energy of the nanospheres converges faster to a constant value of $\approx 94\;\mathrm{meV}/{Å}^{2}$. This is a slightly higher value than γ of any low-index facet (cnf. Table 1) reflecting the fact that a spherical surface composes (from the atomistic point of view) of a large number differently orientated facets. Places where these facets meet (i.e., edges) are composed of atoms with the same or higher number of broken bonds than atoms in the surrounding planar facets, thus, further increasing the surface energy.

#### 3.2.3. Cuboctahedrons

**Figure 4:**
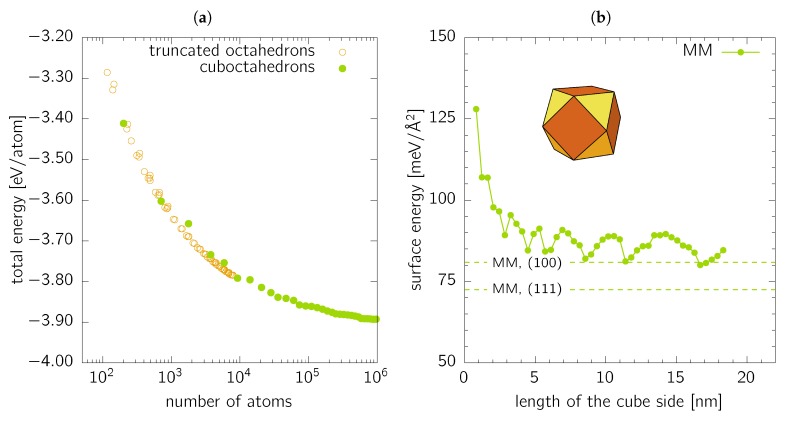
(**a**) Total energy per atom as a function of the nanoparticle size (in terms of number of forming atoms) for cuboctahedrons (full circles) and general truncated octahedrons (open circles). (**b**) Surface energy of cuboctahedrons calculated by MM and showed as a function of the size of “parent” cube. The dashed lines are the MM values for single-orientated (100) and (111) surfaces as listed in Table 1.

The last class of objects studied in this work are cuboctahedrons, i.e., (100)-faceted cubes with all apexes cut by (111) planes (see inset in Figure 4b). Cuboctahedrons are a special subset of truncated octahedrons with the all sites equally long. Figure 4a shows the total energy per atom plotted against the nanoparticle size in terms of the number of forming atoms for cuboctahedrons together with more general truncated octahedrons. The latter were generated with a build-in function of Atomic Simulation Environment toolkit [39] for various sizes of the truncated octahedron apexes. Obviously, the cuboctahedrons are not always the most convenient shape for a given number of atoms.

The surface energy of cuboctahedrons (Figure 4b) oscillates between two values, ≈78 and $\approx 90\;\mathrm{meV}/{Å}^{2}$. This behaviour is caused by the changing ratio of surface atoms forming the (100) and (111) facets and the edges and corners, which directly corresponds with the atomistic nature of the nanoparticle. A detailed analysis of the coordination of the surface atoms reveals that the number of 9-coordinated surface atoms, corresponding to ideal (111) facets, is in anti-phase with the surface energy as shown in Figure 4b. The 8-coordinated (100) surface atoms also show small steps hence causing a non-monotonous increase of their number as a function of the cuboctahedron size. At the same time, the numbers of 10-, 7-, 6-, and 5-coordinated surface atoms forming edges and corners (i.e., atoms with even smaller coordination and, consequently, more broken bonds than those on ideal (100) and (111) facets, and hence increasing the overall surface energy), exhibit the same “oscillations” concerning the cuboctahedron size as the surface energy itself. Therefore, the oscillations are expected to decrease with increasing cuboctahedron size. It is interesting to note that the two limit values for the surface energies, ≈90 and $\approx 80\;\mathrm{meV}/{Å}^{2}$, represent approximately the same range as the two values, 80.9 and $72.5\;\mathrm{meV}/{Å}^{2}$ for pure (100) and (111) facets, respectively. Similarly to the case of nanospheres, the values are somewhat higher than the ideal single-orientated facets due to the presence of the edges and corners.

## 4. Discussion

### 4.1. Correction of the Surface Area for Electronic Cloud

The surface areas calculated in the previous parts corresponds to the convex hull of ionic positions. In our recent paper [13] dealing with predicting surface energy of Au${}_{55}$ cluster, we have discussed the error made by neglecting extend of the electronic cloud. There, a radius correction of 1.3–1.4Å has been proposed under the assumption that the mass density of the nanocluster is the same as that of bulk fcc-Au. Note that, radius corrections of 0.5–0.8Å have been proposed by de Heer [40].

In order to see how neglecting the electronic cloud layer actually influences the predicted surface energies, we re-evaluate the surface areas. Let $\left\{{\vec{R}}_{i}\right\}$ be a set of the atomic (ionic) positions defined with respect to the nanoparticle centre of mass, i.e.,

(4) $$\sum_{i}{\vec{R}}_{i}=\vec{0}\;,$$

where the sum is performed over all atoms in the nanoparticle. Subsequently, a new set of coordinates, $\left\{{\vec{\tilde{R}}}_{i}\right\}$, is defined as

(5) $${\vec{\tilde{R}}}_{i}=\left(|{\vec{R}}_{i}|+\Delta \right){\vec{R}}_{i}^{0}$$

where ${\vec{R}}_{i}^{0}={\vec{R}}_{i}/\left|{\vec{R}}_{i}\right|$ is a unit vector along the direction of ${\vec{R}}_{i}$. This means that all atoms, and in particular those on the convex hull envelope, are shifted by Δ away from the nanoparticle centre of mass. A new surface area is calculated as a convex hull of $\left\{{\vec{\tilde{R}}}_{i}\right\}$ positions for several representative values of Δ.

**Figure 5:**
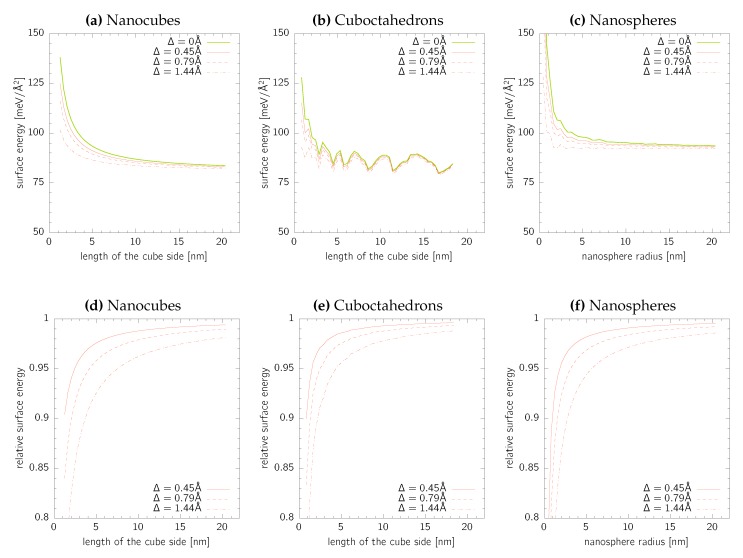
Corrected absolute (upper row) and relative values (lower row) of the surface energies for (**a**,**d**) nanocubes, (**b**,**e**) cuboctahedrons, and (**c**,**f**) nanosheres. The relative surface energies are calculated with respect to the values without correction for the electronic cloud thickness (Δ = 0).

The results are summarised in Figure 5 for all three nanoparticle geometries considered in the present work. In all cases, the surface energy decreases with increasing values of Δ, which is a simple consequence of the surface energy definition in Equation (2). It is, however, remarkable to notice that even for the largest nanoparticle sizes the surface energy reduction is still larger than 1% for the DFT-based electron cloud thickness. We, therefore, conclude that, especially for nanoparticles with specific sizes below 5nm, the correction of the surface area due to the electronic cloud is essential. Moreover, it is likely that for the small nanoparticle sizes, the surface energies calculated here are overestimated due to that fact that even a lower energy can be obtained for a different atomic ordering than fcc (e.g., Mackay icosahedrons as in the case of Au${}_{55}$) or even amorphous liquid-like structures [13]. Finally, it is worth noting that the problem of electronic cloud is not an issue in standard calculations of single orientated flat single crystal facets since it does not influence the actual surface area.

### 4.2. Surface Induced Excess Energy

As mentioned above and discussed in the literature, the surface area of nanoparticles is an ill-defined quantity. In order to eliminate this problem, we introduce a new quantity, $E_{\mathrm{excess}}$, expressing the surface-induced excess energy with respect to the bulk energy corresponding to the same number, *N*, of atoms as in the nanoparticle, normalised to 1 atom, as

(6) $$E_{\mathrm{excess}}=\frac{E_{\mathrm{nanoparticle}}-NE_{\mathrm{fcc}-\mathrm{Au}}}{N}\;.$$

**Figure 6:**
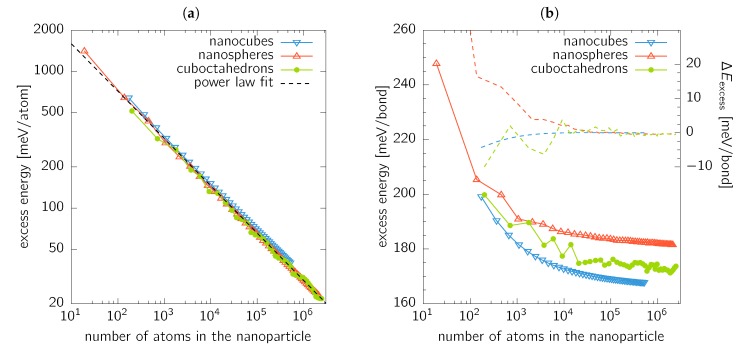
Excess energy, *E*_excess_, of nanoparticles with respect to the bulk fcc Au as a function of the number, *N*, of atoms forming the nanoobject. *E*_excess_ is normalised to (**a**) number of the atoms forming the nanoparticle, and (b) to the number of broken bonds. The dashed lines in (**b**) show the difference between the actual value of *E*_excess_ as calculated by MM, and a fitted value using Equation (10).

A similar concept has been previously demonstrated to work also for energetics of carbon fullerenes [41], or even for elasticity of nanoporous gold [42]. If the excess energy, $E_{\mathrm{excess}}$, is evaluated for nanocubes, nanospheres, and cuboctahedrons, a linear relationship between $\log E_{\mathrm{excess}}$ and logN is obtained independent of the nanoparticle shape (Figure 6a). This suggests that the excess energy is a power law function of the total number of atoms (nanoparticle size). This fit (the dashed line in Figure 6a) gives

(7) $$E_{\mathrm{excess}}=3523.3\;\mathrm{meV}/\mathrm{atom}\times N^{-0.346}.$$

Recalling the idea that the surface energy is genuinely connected with the broken bonds (bb), we now establish the energy needed to “break” a bond. Let us consider an n×n×n nanocube containing atoms with 4 different nearest neighbour coordinations: 8 atoms with 9 bb forming corners (i.e., 3-coordinated atoms), (12n−12) atoms with 7 bb forming the edges (i.e., 5-coordinated atoms), $\left(12n^{2}-12n+6\right)$ atoms with 4 bb forming the surface facets (i.e., 8-coordinated atoms), and $\left(4n^{3}-6n^{2}+3n-1\right)$ bulk atoms with no bb (i.e., fully 12-coordinated atoms). If we simply assume that all bonds “cost” the the same energy $E_{\mathrm{bond}}$ to break them, the excess energy, $E_{\mathrm{excess}}$, i.e., the sum of the contributions described above, follows as

(8) $$E_{\mathrm{excess}}=\left[9\times 8+7\times \left(12n-12\right)+4\times \left(12n^{2}-12n+6\right)\right]E_{\mathrm{bond}},$$

yielding $E_{\mathrm{bond}}=168.1\;\mathrm{meV}/\mathrm{bond}$ from fitting the nanocubes data.

However, the red triangles in Figure 6b, showing the nanocubes excess energy normalised to the number of broken bonds, clearly exhibit a non-constant value for $E_{\mathrm{bond}}$. Consequently, we propose a slightly modified description in which the energy needed to break a bond is a (non-linear) function of the coordination. Hence, it costs different energy to create, e.g., a corner atom (9 broken bonds) than a facet atom (4 broken bonds). Thus the excess energy becomes

(9) $$E_{\mathrm{excess}}=72E_{\mathrm{corner}}+84\left(n-1\right)E_{\mathrm{edge}}+24\left(2n^{2}-2n+1\right)E_{\mathrm{facet}}\;.$$

Fitting yields $E_{\mathrm{corner}}=272.1\;\mathrm{meV}/\mathrm{bond}$, $E_{\mathrm{edge}}=215.2\;\mathrm{meV}/\mathrm{bond}$, and $E_{\mathrm{facet}}=166.0\;\mathrm{meV}/\mathrm{bond}$. It turns out that for nanocubes with side ⪆5nm, Equation (9) provides predictions with an accuracy better than ≈1meV/bond. Energy of a broken bond, corresponding to an infinitely large (100) facet, can be estimated from the surface energies as given in Table 1. This value is 167.5meV/bond, which is close to $E_{\mathrm{bond}}=168.1\;\mathrm{meV}/\mathrm{bond}$ (Equation (8)) as well as $E_{\mathrm{facet}}=166.0\;\mathrm{meV}/\mathrm{bond}$ (Equation (9)).

The complex shapes of cuboctahedrons and nanospheres somewhat restrict the intuitive analysis of the excess energy above presented. When the excess energy is fitted with a single valued energy per broken bond (equivalent to Equation (8)), values of 172.8meV/bond and 181.9meV/bond are obtained for cuboctahedrons and nanospheres, respectively. These values represent an excellent estimation of the excess energies in the limit of large nanoparticles, as shown in Figure 6b. Moreover, the excess energy value for cuboctahedrons lies between the values estimated for (100) ($E_{\left(1\;0\;0\right)}=167.5\;\mathrm{meV}/\mathrm{bond}$) and (111) ($E_{\left(1\;1\;1\right)}=173.3\;\mathrm{meV}/\mathrm{bond}$) facets. This fact further illustrates that the surface energy values, as presented in Section 3.2, are remarkably influenced by the evaluation of the actual surface area (which is, from the atomistic point of view, ill-defined). Consequently, the mean value of the surface energy of cuboctahedrons as shown in Figure 4b lies outside the range bounded by $\gamma_{\left(1\;0\;0\right)}$ and $\gamma_{\left(1\;1\;1\right)}$ values.

Finally, in order to obtain a non-constant behaviour, we fit the excess energy with

(10) $$E_{\mathrm{excess}}=\sum_{i=1}^{11}\left(12-i\right)N\left(i\right)E\left(i\right)$$

where N(i) is the number of *i*-coordinated atoms (i.e., those having (12−i) broken bonds) and E(i) is the corresponding excess energy contribution. Equation (10) is a generalised formulation of Equation (9) reflecting that all possible coordinations may occur due to the shape of nanoparticles. We note that the smallest coordination obtained was 3 and 4 for the case of cuboctahedrons and nanospheres, respectively. The fitted values of E(i) are given in Table 2, and the difference between the actual $E_{\mathrm{excess}}$ from MM and values predicted using Equation (10) is shown in Figure 6b with dashed lines. Obviously, the fit provides excellent agreement for nanoparticles containing $\approx 10^{4}$ atoms and more.

Our analysis provides an insight into the here predicted trends. Regardless of the nanoparticle shape, the surface energy decreases with the increasing particle size. The reason is that the smaller is the nanoparticle, the larger is the fraction of the surface atoms with small coordination, i.e., those with lots of broken bonds. Moreover, the energy to break a bond increases (generally) with the decreasing atom coordination.

### 4.3. Contribution of Surface Stress State

As it has been recently stressed out [43], the excess energy due to a free surface has two contributions: the surface energy contribution related to the energy penalty of broken bond and the contribution due to the elastic strain energy generated by the surface stress state. The latter depends on the surface curvature. As an illustrative example let us assume a spherical body and a homogeneous surface stress state with the value σ leading to a pressure with value 2σ/R in the whole spherical body. From this description it becomes clear that the energetic surface stress contribution is zero for the slab approach. Similarly, the energetic surface stress contribution will be negligible for rather large nanocubes with only a marginal amount of corner and edge atoms (see discussion in the Section 4.2).

We now try to estimate the energetic surface stress contribution to the excess energy for the case of a spherical nanoparticle using classical continuum mechanics. Let us denote *R* the nanosphere’s radius, and γ its surface energy. Furthermore, let us keep to the reasonable assumption that the value of σ and γ are of the same order of magnitude. The corresponding total surface energy is then

(11) $$E_{\gamma }=4\pi R^{2}\gamma .$$

For sake of simplicity, we further assume isotropic elastic properties of the nanoparticle, with ν and *E* being its Poisson’s ratio and Young’s modulus, respectively. The elastic strain energy caused by the surface stress σ, activating an internal pressure 2σ/R, is

(12) $$E_{\sigma }=\frac{4}{3}\pi R^{3}\frac{6(1-2\nu )}{E}\frac{\sigma^{2}}{R^{2}},$$

for details, see, e.g., Ref. [1], Appendix 3. The ratio of the energetic surface stress contribution to the surface energy follows with σ=γ as

(13) $$\frac{E_{\sigma }}{E_{\gamma }}=\frac{2(1-2\nu )}{E}\frac{\gamma }{R}.$$

Taking a representative values for gold, $\gamma =1\;J/m^{2}$, E=78GPa, ν=0.44, and R=1nm, Equation (13) yields $0.359\times 10^{-2}$, i.e., the energetic surface stress contribution to the total excess energy is less than 1% of the surface induced excess energy. This ratio becomes even smaller (negligible) for larger nanospheres.

**Figure 7:**
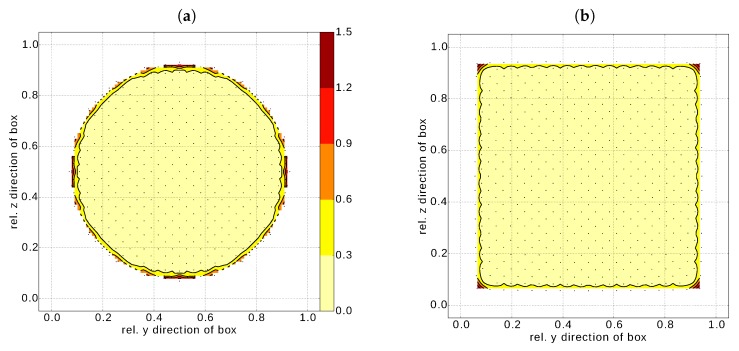
Contour plots of the distribution of the surface stress induced excess energy (in eV/at.) contribution for a cross section of (**a**) a nanoshere (*R* = 3.25 nm) and (**b**) a nanocube (*a* = 6.92 nm). Both cross sections include the nanoparticle centre. The dots represent actual atoms in the cross section, e.g., real locations, where the excess energy is stored. For sake of clear demonstration, the discrete data were interpolated over the whole cross sectional area.

To corroborate this rather simplistic estimation, we plot the excess energy distribution over a cross section including the centre for a nanosphere (Figure 7a) and a nanocube (Figure 7b) as obtained from the MM simulations. Several observations can be made. Firstly, the excess energy is concentrated at the nanoparticle surface irrespective of its shape. The surface stress (and hence the corresponding elastic strain energy) could be only of relevance for a nanosphere. However, we can conclude that this contribution is effectively zero (or negligible). A similar situation can be expected for a nanocube, where the excess energy is concentrated to the nanocube edges (corner of the cross section in Figure 7b). This fact nicely agrees with the fitted values of $E_{\mathrm{edge}}=215.2\;\mathrm{meV}/\mathrm{bond}$ being larger than $E_{\mathrm{facet}}=166.0\;\mathrm{meV}/\mathrm{bond}$, estimated in Section 4.2.

Even though the term surface energy was used in a slightly imprecise way throughout the Section 3.2 (more accurate would be to talk about surface induced excess energy), we conclude that the energy contribution of surface stress can be neglected and the two quantities, surface energy and surface induced excess energy, are equivalent (or at least of the same order of magnitude) for practical cases with nanoparticles larger than ≈1nm.

## 5. Conclusions

A molecular mechanics study, complemented by first principles Density Functional Theory calculations, was performed to obtain surface energy of small gold nanoclusters of various sizes and (geometrically well defined) shapes. The employed interatomic pair potential was shown to give structural parameters and surface energies comparable with DFT-LDA calculations. The surface energy of nanocubes and nanospheres has been shown to converge to a constant value. The convergence was faster in the case of nanospheres compared with nanocubes. The surface energy, γ, is practically constant for any particles with radius larger than ≈3nm. Truncated cubes (cuboctahedrons) did not achieve a single value for the surface energy within the studied range of nanoparticle sizes but, instead, an oscillating behaviour between two values. The range of these oscillations equals to the difference between γ of (100) and (111) facets. Finally, the surface-induced excess energy obviously follows a universal power-law dependence on the number of atoms forming the nanoparticle and is, to a large extent, related to the number of broken bonds (reduced coordination of the surface atoms). Importantly, the size-dependence of surface energy becomes significantly reduced when the actual surface area is corrected by the thickness of the electronic cloud, leading to almost constant values particularly for nanocube and nanosphere sizes of about 5nm and more.

As outlined above, this study has found an increase of the surface energy with decreasing particle size (which is in agreement with other theoretical studies). Two remarks may be useful in this regard. Firstly, this fact should not be confused with experimental works on liquid solution–solid nanoparticle interface energies of gold nanoparticles, moreover often having irregular shapes or even liquid-like surface layer. Secondly, we note that small nanoparticles, specifically the Au${}_{55}$, were shown to be amorphous rather than crystalline. Hence the values predicted here for the smallest particle sizes of a few nanometers are not relevant for amorphous or glassy particles.

In conclusion, this work contributes to understanding of surface energy (solid phase–vacuum interface) of crystalline nanoparticles and its relation to the their structure.

## Figures and Tables

**Table 1:** Calculated surface energies for three low-index facets, including data from the literature for comparison.

	(100)		(110)		(111)	
	[meV/${Å}^{2}$]	[J/m${}^{2}$]	[meV/${Å}^{2}$]	[J/m${}^{2}$]	[meV/${Å}^{2}$]	[J/m${}^{2}$]
DFT-GGA (this work)	54.5	0.87	57.0	0.91	45.2	0.72
DFT-GGA (Ref. [35])					50	0.80
FCD-GGA ${}^{†}$ (Ref. [33])	101.5	1.63	106.1	1.70	80	1.28
MM (this work)	80.9	1.30			72.5	1.16
DFT-LDA (this work)	83.5	1.34	89.2	1.43	78.4	1.26
DFT-LDA (Ref. [35])					80	1.28
experiment (Ref. [36])					93.6	1.50
experiment (Ref. [37])					94.0	1.51

${}^{†}$ FCD = full charge density.

**Table 2:** Fitted coefficients E(i) for the excess energy expression according to Equation (10). The index *i* expresses the coordination of atoms (i.e., (12−i) is the number of broken bonds, bb).

	Nanocubes	Cuboctahedrons	Nanospheres
E(3) [meV/bond]	272.1	287.3	0
E(4) [meV/bond]	0	161.1	426.3
E(5) [meV/bond]	215.2	243.4	258.3
E(6) [meV/bond]	0	163.1	232.0
E(7) [meV/bond]	0	239.5	212.2
E(8) [meV/bond]	166.0	170.3	181.1
E(9) [meV/bond]	0	162.2	159.2
E(10) [meV/bond]	0	93.6	100.7
E(11) [meV/bond]	0	16.9	46.0
